# Case report: Grönblad-Strandberg syndrome

**DOI:** 10.22336/rjo.2022.31

**Published:** 2022

**Authors:** Cătălin Vasile Cărăuș, Alina Maria Spînu, Alexandra Andreea Negrii

**Affiliations:** * Opticlinic Med, Cluj-Napoca, Romania; ** S-MAedica Clinic, Abrud, Romania; *** Department of Ophthalmology, County Emergency Hospital Cluj-Napoca, Romania

**Keywords:** Grönblad-Strandberg syndrome, angioid streaks, Pseudoxanthoma elasticum, choroidal neovascularization

## Abstract

**Objective:** To present a case of secondary type 2 choroidal neovascularization (CNV) and exudative maculopathy in a patient with Grönblad-Strandberg syndrome.

**Methods:** A 37-year-old male was admitted with bilateral progressive painless visual acuity loss and metamorphopsias. A thorough ophthalmologic and clinical examination was performed.

**Results:** Best-corrected visual acuity (BCVA) on presentation was 20/ 200 OD (Oculus Dexter) and 20/ 60 OS (Oculus Sinister). Fundus examination revealed angioid streaks and subretinal hemorrhages on OU (Oculus Uterque), macular fibrosis on OD and “peau d’orange” pigmentary mottling on OS. Leakage areas on fundus fluorescein angiography (FFA) revealed active CNV on OU, which was confirmed by Optical Coherence Tomography (OCT). The presence of typical “plucked chicken” skin lesions in the latero-cervical area and their biopsy confirmed the diagnosis of Pseudoxanthoma elasticum (PXE). Consequently, the diagnosis of Grönblad-Strandberg syndrome was established.

**Conclusions:** Every new diagnosis of angioid streaks entails not only a thorough ophthalmologic evaluation for secondary sight-threatening complications, but also a multidisciplinary evaluation due to the possibility of severe underlying systemic disease.

**Abbreviations:** BM = Bruch’s membrane, RPE = Retinal Pigmented Epithelium, PXE = Pseudoxanthoma Elasticum, ABCC6 = ATP binding cassette subtype C number 6, CNV = Choroidal Neovascularization, BCVA = Best-Corrected Visual Acuity, OD = Oculus Dexter, OS = Oculus Sinister, OU = Oculus Uterque, FFA = Fundus Fluorescein Angiography, OCT = Optical Coherence Tomography, IPO = Intraocular Pressure, ECG = Electrocardiogram, anti-VEGF = anti-vascular endothelial growth factor

## Introduction

Angioid streaks represent visible breaks in a calcified and thickened Bruch’s membrane (BM), which are associated with atrophic degeneration of the overlying retinal pigmented epithelium (RPE) and the absence of the underlying choriocapillaris. The etiology is extremely varied but regardless the systemic or ocular associations, the pathological mechanism is similar: calcium binding followed by disintegration and frying of the elastic fibers from the midsegment of BM [**1**]. 

Although considered the most prevalent, the association of angioid streaks and Pseudoxanthoma elasticum (PXE), also known as Grönblad-Strandberg syndrome, remains a rare clinical finding. While the estimated prevalence of PEX is between 1/ 25.000 and 1/ 100.000, only 59-87% of cases develop angioid streaks. There is an unexplained female predominance (2:1) [**2**,**3**]. Over 110 different mutations with autosomal recessive inheritance affect the ABCC6 (ATP binding cassette subtype C number 6) gene and induce elastorrhexis - mineralization and fragmentation of elastic fibers [**3**]. This high degree of allelic heterogeneity translates into marked phenotypical heterogeneity regarding the extent and severity of organ system involvement (integumental, cardiovascular or ocular) [**4**]. Other ocular manifestations in PEX include “peau d’orange” pigmentary mottling, subretinal or intraretinal hemorrhages, choroidal neovascularization (CNV), optic nerve drusen and comet-shaped lesions [**5**].

## Case report

A 37-year-old male presented to the ophthalmology department with bilateral painless visual acuity loss and metamorphopsias. The symptoms had an insidious onset approximatively 1 year before presentation and progressive evolution, but an exacerbation in the previous days prompted him to seek medical advice. The family history revealed stage II hypertension (mother) and ischemic stroke (father), but the patient’s medical history was noncontributory. 

**Fig. 1:**
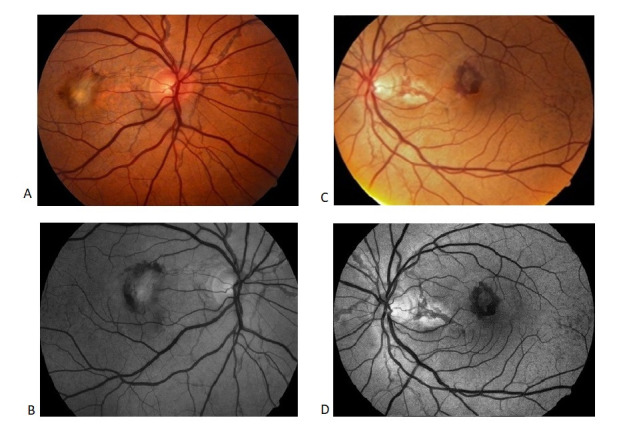
Color and red-free retinophotography Angioid streaks radiating from the optic nerve associated with a fibrotic macular plaque of approximatively 1.5-disc diameters, surrounded by subretinal hemorrhage OD (A, B); angioid streaks radiating from the optic nerve associated with a macular subretinal hemorrhage and temporal “peau d’orange” pigmentary mottling OS (C, D)

**Fig. 2:**
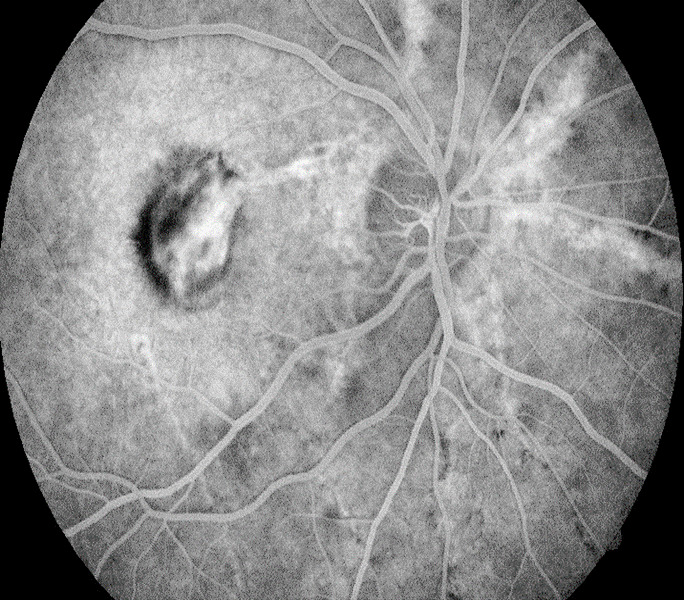
Fundus Fluorescein Angiography of OD: venous time Hyperfluorescent linear lesions radiating from the optic nerve (angioid streaks) associated with a diffuse macular area of intense hyperfluorescence caused by leakage from choroidal neovascularization and staining of subretinal fibrosis; superotemporal hypofluorescent area caused by the masking effect of a subretinal hemorrhage

**Fig. 3:**
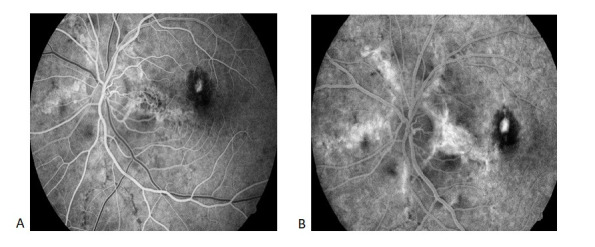
Fundus Fluorescein Angiography of OS Hyperfluorescent linear lesions radiating from a peripapillary ring (angioid streaks) associated with a macular area of hyperfluorescence surrounded by a hypofluorescent area of subretinal hemorrhage can be seen during both the arterial and venous time (A, B); enlargement of the macular area of hyperfluorescence due to leakage can be observed during the venous time (B)

**Fig. 4:**
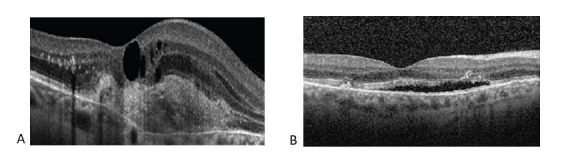
Optical Coherence Tomography: horizontal macular sections Type 2 choroidal neovascularization proliferating into the subretinal space between the neurosensory retina and the retinal pigment epithelium associated with macular oedema and intraretinal fluid accumulation OD (A); subfoveal fluid accumulation OS (B)

Best-corrected visual acuity (BCVA) was 20/ 200 OD (Oculus Dexter) and 20/ 60 OS (Oculus Sinister) after correction of a myopic compound astigmatism (OD: -1,00 sf ≈-1,25 cyl x 150°and OS: -1,25 sf ≈-1,00 cyl x 110°). The intraocular pressure (IOP) was 16 mmHg OD and 17.5 mmHg OS (Goldman Applanation Tonometer). Pupillary response and extraocular motility were normal, as well as anterior segment examination. Fundus examination revealed the presence of linear irregular red-brown lesions, radiating from the optic nerve OU. A fibrotic macular plaque surrounded by subretinal hemorrhage was observed on the OD, and a macular subretinal hemorrhage and temporal “peau d’orange” pigmentary mottling on the OS (**Fig. 1**) On Fundus Fluorescein Angiography (FFA), the linear lesions mentioned previously were hyperfluorescent due to the window defect associated with breaks in BM and atrophy of the overlying RPE, aspect that confirmed the diagnosis of angioid streaks (**Fig. 2**, **3**). The presence of macular areas of late leakage OU confirmed the presence of active CNV. Optical Coherence Tomography (OCT) examination (SD-OCT, Revo NX, Optopol Technology Sp. Z.o.o., Zawiercie, Poland) also revealed the presence of type 2 CNV expanding into the subretinal space between the neurosensory retina and RPE, associated with macular oedema OD and subfoveal fluid accumulation OS (**Fig. 4**).

**Fig. 5:**
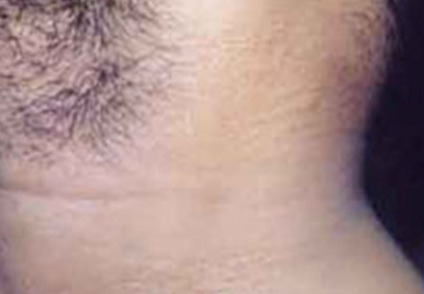
Characteristic skin lesions in patients with Pseudoxanthoma Elasticum Non-follicular yellow-white papules of approximatively 3 mm coalescent in the latero-cervical area

Physical examination revealed the presence of non-follicular yellow-white papules of approximatively 3 mm, coalescent in the left latero-cervical area (**Fig. 5**). The lesions were asymptomatic and the skin elasticity was preserved. No other lesions were observed in the flexural areas. This characteristic aspect of “plucked chicken” skin prompted a skin biopsy, which revealed calcification and disruption of elastic fibers in the deep dermis, confirming the diagnosis of PEX [**5**].

An extensive laboratory evaluation including complete blood count, coagulation tests, liver, and kidney function, glycemia, electrolytes, lipid profile and inflammatory markers, was performed. Except for dyslipidemia (cholesterol=223 mg/ dL, triglycerides=295 mg/ dL), no pathological findings were noted. Cardiologic evaluation revealed symmetrical palpable peripheral pulses, no murmurs, and a normal blood pressure of 135/ 80 mmHg. ECG (Electrocardiogram) was normal with sinus rhythm, a heart rate of 65 bpm, intermediary QRS and no ST-T alterations. Normal structure and function were also confirmed by cardiac echography. Gastroenterological evaluation including an abdominal echography identified no abnormalities. Tibial and sacrococcygeal radiographies revealed no visible alterations, while the cervicothoracic spine radiography revealed only a minimum thoracic levoscoliosis. 

Considering all the clinical and ancillary examinations performed, we established the diagnosis of Grönblad-Strandberg syndrome, which associates PXE with angioid streaks, complicated in our case by secondary type 2 CNV and exudative maculopathy OU. 

The local treatment consisted of 3 consecutive intravitreal anti-vascular endothelial growth factor (anti-VEGF) injections (Bevacizumab) administered off-label at a dose of 1.25 mg/ 0.005 mL per injection at 4 weeks interval. The systemic treatment included oral supplements with lutein and zeaxanthin for 3 months and a diet rich in vegetables, fruits, and fish. 

**Fig. 6:**
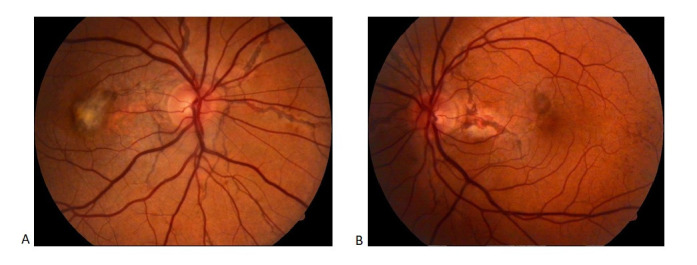
Color retinophotography at 6 months Angioid streaks radiating from the optic nerve associated with a fibrotic macular plaque of approximatively 1.5-disc diameters OD (A); angioid streaks radiating from the optic nerve associated with a small fibrotic lesion at the superonasal macula and temporal “peau d’orange” pigmentary mottling OS (B)

**Fig. 7:**
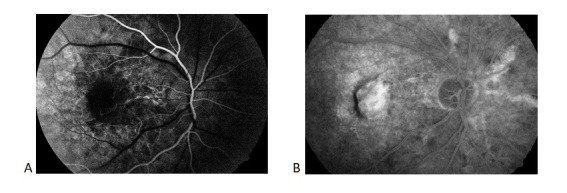
Fundus Fluorescein Angiography OD at 6 months Hyperfluorescent linear lesions radiating from the optic nerve (angioid streaks) can be seen during both arterial and venous time (A, B); a macular hypofluorescent area caused by fibrosis can be seen during arterial time (A); the same lesion becomes intensely hyperfluorescent during venous time due to staining

**Fig. 8:**
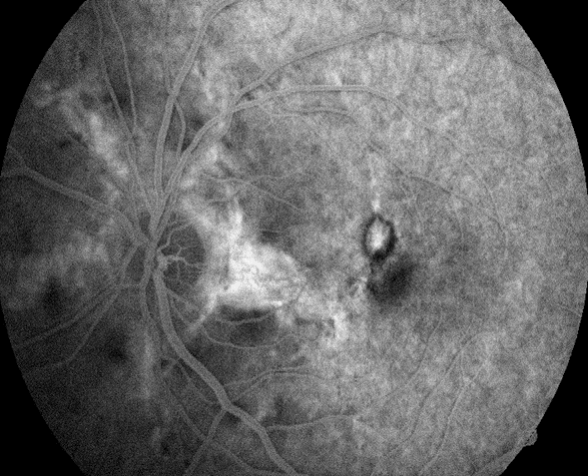
Fundus Fluorescein Angiography OS: venous time Hyperfluorescent linear lesions radiating from a peripapillary ring (angioid streaks) associated with a small area of hyperfluorescence at the superonasal macula, surrounded by a hypofluorescent ring due to subretinal fibrosis

**Fig. 9:**
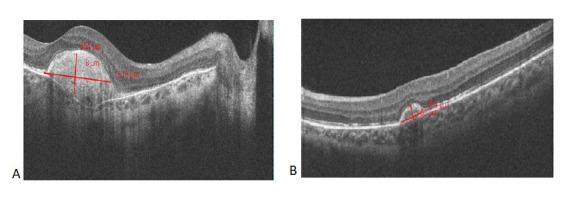
Optical Coherence Tomography: horizontal section Fibrosis of choroidal neovascularization OD (A) and OS (B)

The evolution was favorable. At 6 months, the BCVA improved to 20/ 25 OD and 20/ 20 OS. Fundus examination revealed remission of subretinal hemorrhages and fibrosis of previously active lesions (**Fig. 6**). Fibrosis of CNV and absence of activity were confirmed by FFA and OCT (**Fig. 7**-**9**). OCT also revealed the resolution of intraretinal (OD) and subretinal (OS) fluid (**Fig. 9**).

## Discussion

The differential diagnosis in our case had to be made with other ocular pathologies mimicking angioid streaks, including choroidal ruptures and lacquer cracks, as well as with other secondary causes of CNV, including the diseases mentioned previously, but also exudative age-related macular degeneration. A multidisciplinary evaluation was conducted to identify any systemic diseases associated with the angioid streaks, and to assess the extent of organ system involvement once the diagnosis of PEX has been established.

Angioid streaks can be associated with secondary sight-threatening complications: traumatic breaks in BM, subretinal and intraretinal hemorrhages, macular atrophy and CNV. Male sex, longer angioid streaks and longer evolution of disease have been associated with higher risk of developing CNV and macular atrophy [**6**]. Patients with PXE have a higher risk of macular degeneration secondary to CNV compared to other systemic associations, and therefore have a worse visual prognosis [**3**].

There is no specific treatment available for PXE or angioid streaks. Current management of the disease includes secondary prevention by avoiding ocular traumas and periodic monitoring for the development of CNV, the only situation that entails the need of active treatment. The most efficient therapy available is the intravitreal administration of anti-VEGF, which stops the progression of CNV with both functional (BCVA) and morphological (reduction in leakage and retinal oedema) improvement. To achieve maximal efficiency, the treatment of CNV in PXE must be initiated as soon as possible because any delay can cause sustained decrease in vision [**7**]. Although in our case the patient presented to the doctor a year after the onset of symptoms, the evolution after 3 intravitreal anti-VEGF injections was favorable and the results were comparable with those reported by Finger et al [**7**]. Often, PXE patients have an increased cardiovascular risk profile, therefore careful consideration of the possible complications of intravitreal administration of Bevacizumab must be considered before treatment initiation [**3**,**7**]. In our case, the cardiologic evaluation excluded any functional or structural alterations and therefore treatment initiation was possible.

Relapses are possible and consequently the need for constant screening and monitoring with timely intervention is essential for visual rehabilitation. However, there are no guidelines regarding the type and rhythm of follow up [**5**,**7**]. Unlike the case of exudative age-related macular degeneration, the periodic administration of intravitreal anti-VEGF in angioid streaks is not applied because trauma and pressure induced by the injection can cause extension of streaks. Nevertheless, this association remains controversial [**3**].

Cardiovascular complications can be fatal, but most patients with PXE have a normal life span. Cardiovascular involvement can cause mucosal bleeding or occlusive arterial disease with peripheral arteritis, digestive angina, coronary artery disease and cerebrovascular disease [**4**]. Despite the association of severe additional impairment, visual impairment has the greatest impact on disability and quality of life in patients with Grönblad-Strandberg syndrome [**5**,**8**]. 

## Conclusion

The aim of this case report was to highlight the importance of a complete ophthalmologic evaluation once a new diagnosis of angioid streaks has been established due to the possibility of secondary sight-threatening complications. A multidisciplinary evaluation for systemic underlying diseases is also mandatory for all patients on first examination, regardless of the presence or absence of symptoms, to ensure proper treatment and monitoring, and therefore reduce morbidity and mortality.

**Conflict of interest**

The authors state no conflict of interest.

**Informed Consent and Human and Animal Rights statement**

An informed consent was obtained from the patient included in the case report.

**Acknowledgements**

None.

**Sources of Funding**

None.

**Disclosures**

None.
